# Stac3 Is a Novel Regulator of Skeletal Muscle Development in Mice

**DOI:** 10.1371/journal.pone.0062760

**Published:** 2013-04-23

**Authors:** Brad M. Reinholt, Xiaomei Ge, Xiaofei Cong, David E. Gerrard, Honglin Jiang

**Affiliations:** Department of Animal and Poultry Sciences, Virginia Polytechnic Institute and State University, Blacksburg, Virginia, United States of America; Mayo Clinic, United States of America

## Abstract

The goal of this study was to identify novel factors that mediate skeletal muscle development or function. We began the study by searching the gene expression databases for genes that have no known functions but are preferentially expressed in skeletal muscle. This search led to the identification of the Src homology three (SH3) domain and cysteine rich (C1) domain 3 (*Stac3)* gene. We experimentally confirmed that *Stac3* mRNA was predominantly expressed in skeletal muscle. We determined if *Stac3* plays a role in skeletal muscle development or function by generating *Stac3* knockout mice. All *Stac3* homozygous mutant mice were found dead at birth, were never seen move, and had a curved body and dropping forelimbs. These mice had marked abnormalities in skeletal muscles throughout the body, including central location of myonuclei, decreased number but increased cross-sectional area of myofibers, decreased number and size of myofibrils, disarrayed myofibrils, and streaming Z-lines. These phenotypes demonstrate that the *Stac3* gene plays a critical role in skeletal muscle development and function in mice.

## Introduction

Skeletal muscle is essential to human health and well-being and is at the core of the animal production industry. It is responsible for approximately 40 percent of body mass in mammals and provides power for respiration, locomotion, and structural support essential for survival. The basic unit of skeletal muscle is the myofiber, a multinucleated cell created by the fusion of single-nucleated muscle progenitor cells, myoblasts. Formation of myofibers from myoblasts (i.e., myogenesis) is chiefly regulated by the myogenic regulatory factors (MRFs), including MyoD, Myf5, myogenin, and MRF4 [1]–[5]. MyoD and Myf5 are required for myogenic lineage determination [5], [6], whereas myogenin and MRF4 regulate differentiation of myoblasts into functional myofibers [5], [7]–[9].

Myogenesis is a complex process. Despite the identification of the aforementioned MRFs, this process is likely controlled by additional factors. To identify novel factors that regulate myogenesis, we searched the gene expression databases for genes that are preferentially expressed in skeletal muscle using the GeneNote program [10]. This search led to the identification of the *Src* homology three (SH3) domain and cysteine rich (C1) domain 3 (*Stac3*) gene. The S*tac3* gene is the third member of the *Stac* gene family that also includes *Stac* and *Stac2* [11]. Unlike *Stac3, Stac* and *Stac2* are expressed specifically in neurons [12]. Prior to the present study, no function had been associated with any member of the *Stac* family.

Because it is expressed predominantly in skeletal muscle and because it is predicted to encode two important functional domains, we hypothesized that *Stac3* might play an important role in skeletal muscle development and function. We tested this hypothesis by disrupting *Stac3* gene transcription in mice. Our data show that *Stac3* is essential for development of functional skeletal muscle and viable mice, and that *Stac3* mutant mouse is a novel mammalian model for myopathies.

## Materials and Methods

### RT-PCR and real-time PCR

Total RNA from mouse tissue samples was isolated with an RNeasy Mini Kit from QIAGEN (Valencia, CA), following the manufacturer's instructions. cDNA was transcribed from total RNA using random primers and the ImProm-II™ reverse transcriptase (Promega, Madison, WI), according to the manufacturer's instructions. PCR and real-time PCR were performed using the PCR Master Mix (Promega) and the SyberGreen PCR Master Mix (Applied Biosystems Inc., Foster City, CA), respectively. In these PCRs, *Gapdh* mRNA or *18s* rRNA was amplified as an internal control. The *Stac3, Gapdh,* and *18s* cDNAs were amplified using the following PCR primers: 5′-TACAGCGACCAACAGTACGC-3′and reverse 5′-TCTGCATTGTTTCCATCCTG-3′; 5-‘ACCCAGAAGACTGTGGATGG-3′ and 5′-GGATGCAGGGATGATGTTCT-3′; and 5′-TTAAGAGGGACGGCCGGGGG-3′ and 5′-CTCTGGTCCGTCTTGCGCCG-3′, respectively. All PCR products were verified by DNA sequencing.

### Generation of *Stac3* mutant mice

*Stac3* mutant mice were generated by the Knockout Mouse Project (KOMP; University of California at Davis) using the knockout-first strategy [13]. Two *Stac3*-targeted (*Stac3* ^tm1a(KOMP)Wtsi^) embryonic stem (ES) clones (EPD0101_1_A09, EPD0101_1_A10) on a C57BL/6N background were injected into blastocysts from C57BL/6 Albino mice. In both clones, the *Stac3* gene is inserted with the “SA-βgeo-pA” trapping cassette between exons 1 and 2, and this insertion is expected to disrupt the generation of *Stac3* mRNA and Stac3 protein. Seven male offspring from injecting the A09 ES cells and one male from injecting the A10 cells were found to be chimeric (30–95%). Two males with 95% and 85% chimerism were selected to breed with C57BL/6 Albino females. The 95% male chimera successfully transmitted the targeted *Stac3* locus to its offspring as evidenced by the coat color (black) and by genotyping (see below). Pairs of mice heterozygous for the targeted *Stac3* allele (*Stac3^+/−^*) were mated to generate *Stac3* homozygous mutant (*Stac3^−/−^)* mice. Mice were housed at designated facility on a timed 12 h light/dark schedule with free access to standard rodent diet and water. All procedures involving animals were approved by the Virginia Tech Institutional Animal Care and Use Committee.

### Genotyping

Genomic DNA was isolated from ear notches or tail clips using a DNeasy Blood & Tissue Kit from QIAGEN. Mouse genotypes were determined by PCR using two pairs of primers. One pair of primers (forward 5′-CTCCATAGCTCTACCGCAGTC-3′ and reverse 5′-CTCTGCCTTGTGAGTGTGGA-3′) was designed to flank the third loxP site in the trapping cassette. This pair of primers was expected to generate a 344 bp PCR product from the targeted *Stac3* allele and a 317 bp product from the wild-type *Stac3* allele. The second pair of primers (forward 5′-TGTTGGGCTTCTTCGTCTCT-3′ and reverse 5′-TGGTACCTTGTGTGGTGGTG-3′) was designed to amplify a 468 bp intron region of the wild-type *Stac3* allele. These PCR reactions were set up using the PCR Master Mix from Promega. Sequences of all PCR products were confirmed by DNA sequencing.

### Histology

Whole mouse fetuses or dissected hind limbs were fixed in 10% neutral buffered formalin for 48 h, embedded in paraffin, and sectioned on a microtome according to standard procedures. Serial 6 μm sagittal sections were cut from the whole body. Serial 6 μm longitudinal and cross sections were cut from the limbs. For limbs, sections were collected until representative sections from the widest girth of the limb were captured. Sections were mounted on Superfrost Plus micro slides (VWR International, Radnor, PA) and were deparaffinized using standard paraffin clearing and rehydration methods. Slides were stained with hematoxylin and eosin (H&E) following standard procedures. Stained sections were photographed using a Nikon eclipse E600 microscope connected to a QColor3 OLYMPUS digital camera.

### Electron microscopy

Transmission Electron Microscopy (TEM) was used to evaluate the ultrastructure of skeletal muscle. Lower leg samples from newborn mice were fixed in 3% glutaraldehyde dissolved in phosphate buffered saline (PBS). The remaining steps were performed by the Morphology Service Laboratory of the Virginia-Maryland Regional College of Veterinary Medicine (Blacksburg, VA), following standard protocols. Both thin (60–90 nm) and thick (5 μm) sections were cut. The thick sections were stained with H&E to verify the location of the extensor digitorum longus (EDL) muscle, and the thin sections were stained with uranyl acetate and lead citrate for electron microscopy. Electron micrographs were captured using a Ziess 10 CA electron microscope at a voltage of 60 KV (Ziess Electron Microscopy, Thornwood, NY) on a AMT 4.10 & PCDIG side mount camera using the AMT V601 Image Capture Engine software (Advanced Microscope Technologies, Woburn, MA).

### Whole-mount β-galactosidase staining

This was done as described previously [14]. Briefly, mouse embryos were fixed in 4% glutaraldehyde dissolved in PBS supplemented with 5 mM EGTA and 2 mM MgCl_2_ for 30 min. Fixed embryos were washed in PBS supplemented with 5 mM EGTA, 2 mM MgCl_2_, 0.01% sodium desoxycholate, and 0.02% Nonidet P-40. To stain for β-galactosidase, embryos were incubated in 0.1% X-gal in PBS supplemented with 2 mM MgCl_2_, 5 mM EGTA, 5 mM K_3_Fe(CN)_6_, 5 mM K_4_Fe(CN)_6_, and 0.01% Nonidet P-40. All steps were performed at room temperature. Pictures of stained embryos were taken with a S2-CTV OLYMPUS anatomical microscope or a Nikon Eclipse 80i microscope connected to a QColor3 OLYMPUS digital camera.

### Immunohistochemistry

Antigen retrieval was performed by boiling slides in 10 mM citrate buffer adjusted to pH 6 at 95°C for 30 min and then cooling them at room temperature for 20 min. Nonspecific binding was blocked by incubating slides with 5% goat serum (Sigma-Aldrich, St. Louis, MO) diluted in PBS for 1 h at room temperature. Myofibers were detected by incubating slides with 1∶200 diluted sarcomeric myosin heavy chain antibody MF20 (Developmental Studies Hybridoma Bank, University of Iowa, Iowa City, IA) and then with 1∶200 diluted DyLight 594-conjugated goat anti-mouse secondary antibody (Fisher Scientific, Pittsburgh, PA). Cell membranes were stained with 1∶400 diluted fluorescein-conjugated wheat germ agglutinin (Invitrogen, Grand Island, NY). Nuclei were stained with DAPI (Invitrogen). Aqueous Prolong Gold antifade reagent mounting medium was applied over the sections for preservation (Invitrogen). Fluorescent images were captured with a Nikon eclipse TS100 connected to a CoolSNAP HQ2 monochrome camera (Photometrics, Tucson, AZ) using a NIS-Elements AR Ver3.1 software program (Nikon Imaging Inc., Melville, NY).

### Quantitative analyses of myofibers

The EDL muscle was used to determine muscle fiber characteristics due to its uniformity and easy recognition. Total number and average cross sectional area of myofibers in an EDL muscle were determined by counting and averaging all myosin heavy chain (MyHC) positive fibers on three serial cross sections at the widest girth of the EDL muscle using the NIS-Elements AR Ver3.1 software.

### Statistical analyses

Data were analyzed using ANOVA followed by Tukey HSD multiple comparison of means in the JMP software (SAS, Cary, NC). A difference was considered statistically significant when *P*<0.05. Data are presented as means ± standard error of the mean (SEM).

## Results

### *Stac3* mRNA was predominantly expressed in skeletal muscle

**Figure 1:**
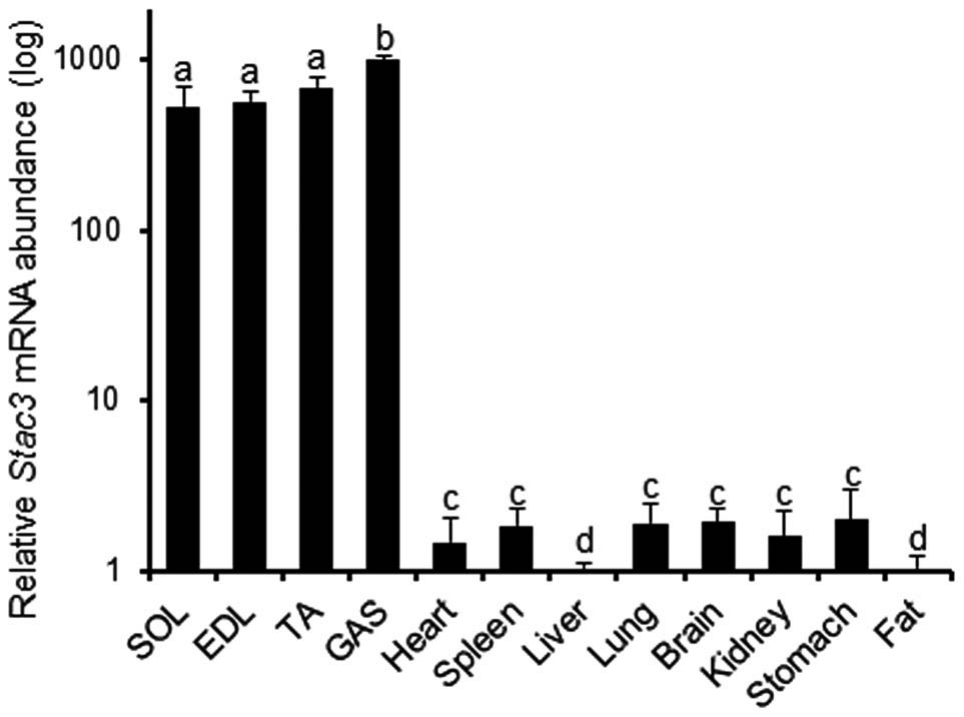
Skeletal muscle-predominant expression of *Stac3* mRNA in mice. Tissues from three adult C57BL/6 male mice were analyzed by real-time RT-PCR. In this analysis, *18s* rRNA was used as an internal control. Data are expressed as mean ± SEM (n = 3). Bars not sharing the same letter labels are different (*P*<0.05). The y-axis is displayed on a log_10_ scale. M: molecular ladder; SOL: soleus; EDL: extensor digitorum longus; TA: tibialis anterior; GAS: gastrocnemius.

Searching the gene expression database at GeneNote with “skeletal muscle” [10], the program generated a list of human genes that were predicted to be specifically expressed in skeletal muscle. This list contained *Stac3*. We became more interested in this gene than the other genes on the list because it had no known function but was predicted to encode a protein containing a SH3 domain and a C1 domain, two classical functional domains [15], [16]. We examined the expression profile of *Stac3* mRNA in adult mice by quantitative RT-PCR. As shown in Fig. 1, *Stac3* mRNA was abundantly expressed in skeletal muscle of four different locations, but was not expressed or expressed at very low levels in any of non-skeletal muscle tissues or organs examined, including brain, heart, and the smooth muscle-containing stomach.

### *Stac3* mutant mice died at birth

**Figure 2:**
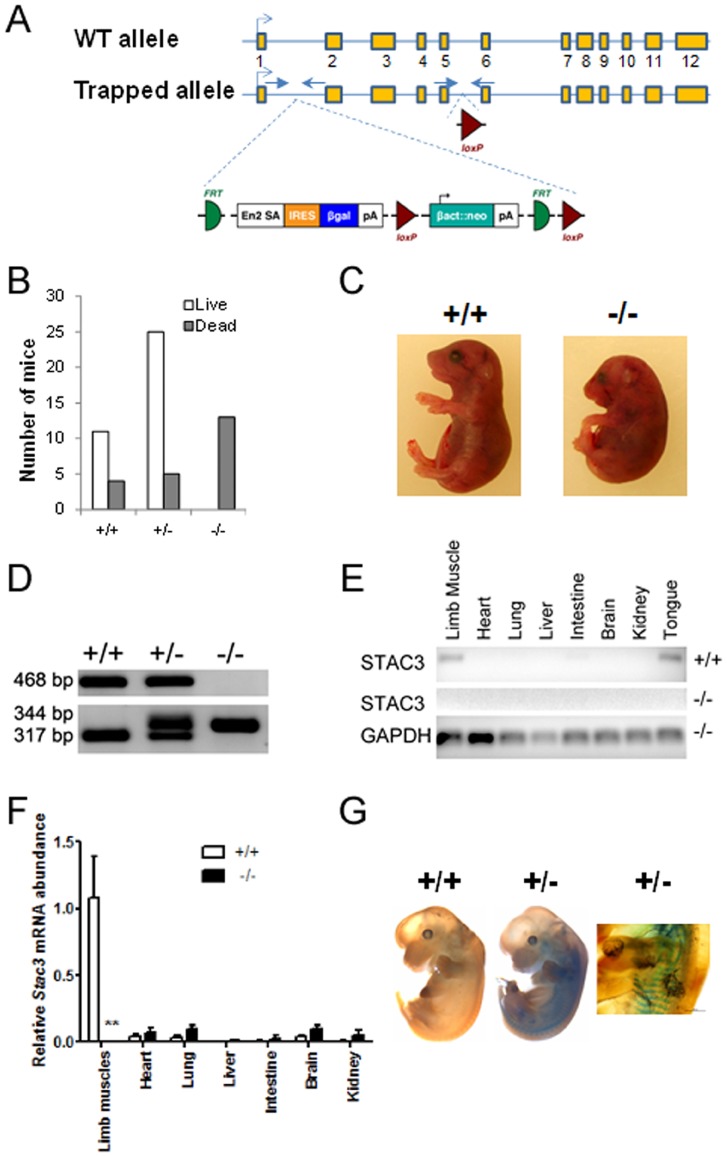
Generation and analyses of *Stac3* mutant mice. **A**, Schematic representation of the targeted *Stac3* allele. The wild-type (WT) *Stac3* allele has 12 exons, with exon 3 containing the translation start codon. The targeted allele is inserted with a trapping cassette between exons 1 and 2, and this insertion is expected to disrupt the generation of normal *Stac3* mRNA. Arrows indicate locations of PCR primers for genotyping. **B**, Genotypes and life of newborn offspring from intercrossing mice heterozygous for the targeted *Stac3* allele. “+/+”, “+/−”, and “−/−” indicate wild-type mice, and mice heterozygous and homozygous for the targeted *Stac3* allele, respectively. A total of 10 litters were included in this analysis. C, Images of a *Stac3* homozygous mutant mouse and a wild-type littermate. Note the curved body shape and dropping forelimbs in the mutant. **D,** Genotyping by PCR. See panel A for the locations of two pairs of primers. **E**, Validation of diminished expression of *Stac3* mRNA in *Stac3* ^−/−^ mice by standard RT-PCR. Shown is a representative image. **F**, Real-time RT-PCR validation of diminished expression of *Stac3* mRNA in *Stac3* ^−/−^ mice. Data are expressed as mean ± SEM (n = 4). ** *P*<0.01 *vs.* “+/+”. **G**, LacZ staining of E13 wild-type and *Stac3* ^+/−^ embryos. Shown are representative images. The image on the right highlights the stained somites in an E13 *Stac3* ^+/−^ embryo.

Mice heterozygous for the targeted *Stac3* allele (*Stac3* ^+/−^) were generated by injecting blastocysts with the *Stac3*-targted ES cells (Fig. 2A). Both male and female *Stac3* ^+/−^mice were viable and fertile and grew normally compared to their wild-type littermates. Breeding between *Stac3^+/^* ^−^ mice resulted in normal-sized litters with a near Mendelian representation of genotypes, but all of the mice homozygous for the targeted *Stac3* allele (*Stac3* ^−/−^) were found dead at birth (Fig. 2B). All *Stac3* ^−/−^ mice had a curved body and dropping forelimbs (Fig. 2C). *Stac3* ^−/−^ fetuses were never seen move and did not respond to touch, but they had heart beating when dissected out of the uterus. Genotypes of *Stac3* ^−/−^ or *Stac3* ^+/−^ mice were confirmed by PCR (Fig. 2D). At E18.5, *Stac3* ^−/−^ fetuses weighed approximately 11% and14% less than their heterozygous and wild-type littermates, respectively (*P*<0.05).

### *Stac3* mRNA expression was markedly diminished in *Stac3* mutant mice

Insertion of the trapping cassette between exon 1 and exon 2 of the *Stac3* allele was expected to disrupt the generation of normal *Stac3* transcript (Fig. 2A). This was confirmed by the fact that *Stac3* mRNA was barely detectable in any tissues examined, including skeletal muscle, in E18.5 *Stac3* ^−/−^ fetuses (Fig. 2E and 2F). As in adult wild-type mice (Fig. 1), *Stac3* mRNA was preferentially expressed in skeletal muscle or skeletal muscle-containing organs such as the tongue in E18.5 wild-type fetuses (Fig. 2E and 2F). The trapping cassette contained a β-galactosidase gene, and this gene was expected to be expressed under the control of the original *Stac3* promoter (Fig. 2A). LacZ staining of E13 embryos confirmed the expected activity of β-galactosidase in somites and limb muscles in *Stac3* ^+/−^ but not in wild-type embryos (Fig. 2G).

### Skeletal muscle of *Stac3* mutant mice had abnormal morphology

**Figure 3:**
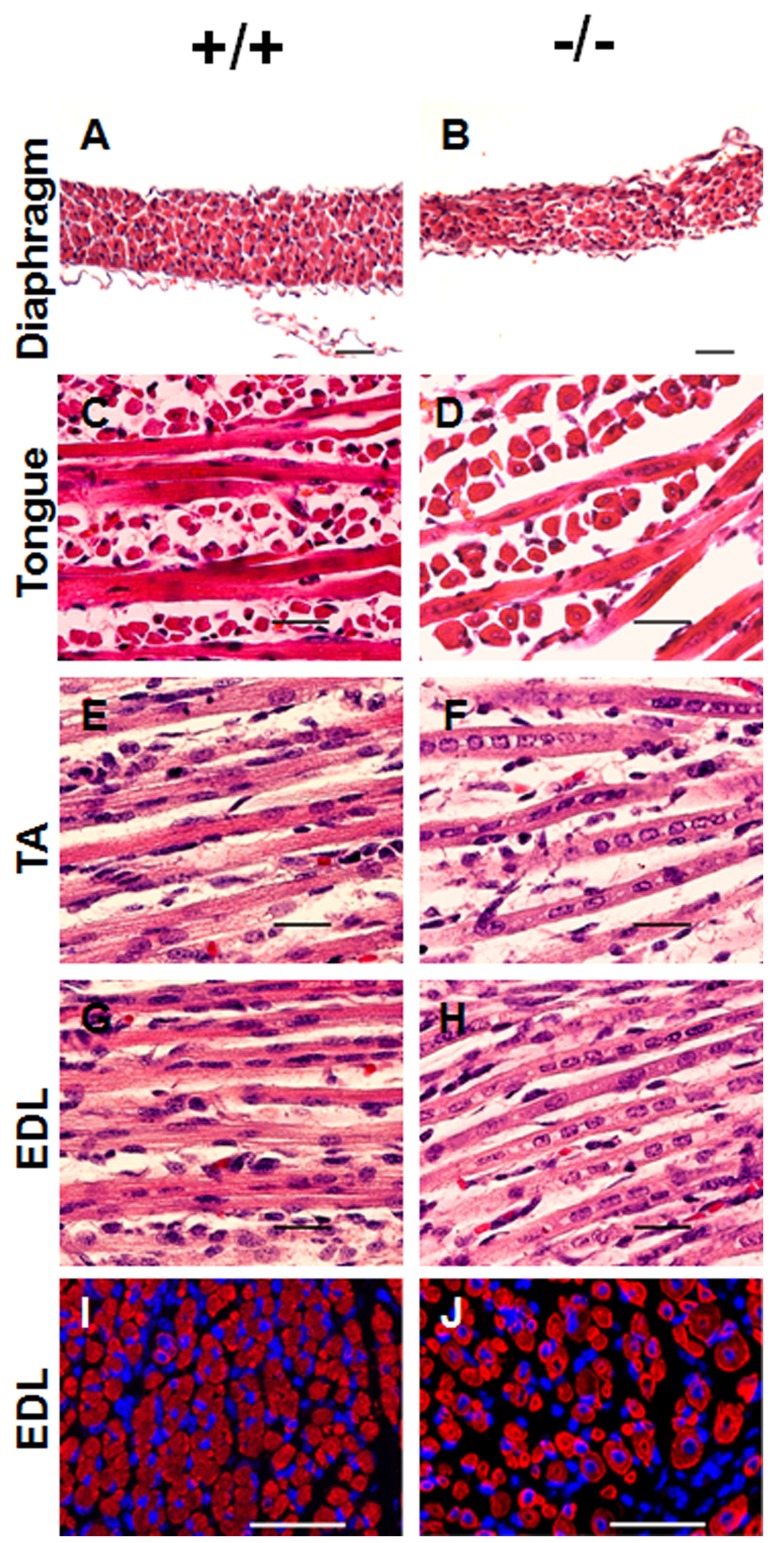
Histological analyses of skeletal muscles in newborn *Stac3* homozygous mutant mice (−/−) and wild-type (+/+) littermates. **A–H**, Hematoxylin and eosin staining of diaphragm, tongue, tibialis anterior (TA), and extensor digitorum longus (EDL) muscles. Note the difference in the location of myonuclei between the two genotypes. **I** and **J**, Immunohistochemical staining of EDL muscle for myosin heavy chain protein (red) and nuclei (blue). Shown are representative images taken from matched areas. Scale bars  = 50 μm for micrographs A, B, I, and J. Scale bars  = 25 μm for micrographs C-H.

**Figure 4:**
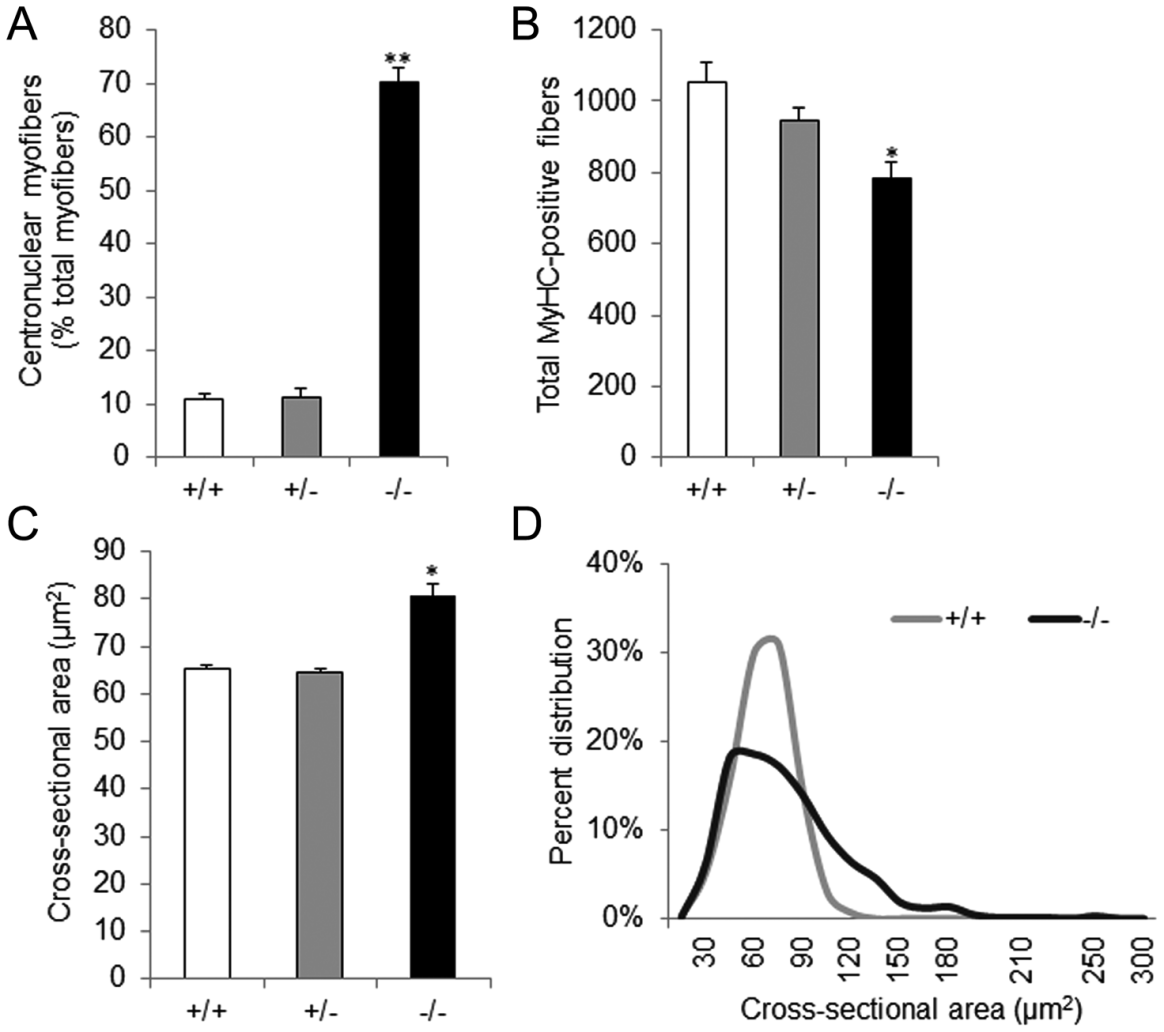
Quantitative analyses of extensor digitorum longus (EDL) muscle in newborn *Stac3* ^−/−^ mice and wild-type and heterozygous littermates. **A**, Percentage of myofibers that had centrally located nuclei. **B**, Total number of myosin heavy chain (MyHC)-positive myofibers at the widest girth of EDL muscle. **C**, Average cross-sectional area of myofibers in EDL muscle. **D**, Frequency distribution of cross-sectional areas (CSA) of myofibers in EDL muscle. Data are expressed as mean ± SEM (n = 3 to 7). * *P*<0.05 and ** *P*<0.01 *vs.* wild-type or heterozygous mice.

Based on gross dissection and H&E staining, *Stac3* ^−/−^ mice had no obvious structural aberrations in viable organs such as heart, liver, kidney, and the gastrointestinal tract, and had no edema or necrosis, disorders that are typically associated with *in utero* death. The diaphragms of newborn *Stac3* ^−/−^ mice were slightly thinner than those of their wild-type littermates (Fig. 3A and 3B). In newborn wild-type mice, most myofibers had peripherally located nuclei (Fig. 3A, 3C, 3E, 3G, and 3I) and were polygonally shaped on cross sections (Fig. 3A, 3C, and 3I). However, in their *Stac3* ^−/−^ littermates, the majority of myofibers had centrally located nuclei (Fig. 3B, 3D, 3F, 3H, and 3J) and appeared round on cross sections (Fig. 3B, 3D, and 3J). Quantitative analyses of the EDL muscle revealed that more than 70% of myosin heavy chain-positive cells in newborn *Stac3* ^−/−^ mice had centrally located nuclei, whereas that number was only 11% in wild-type or heterozygous littermates (*P*<0.01) (Fig. 4A).

### Skeletal muscle of *Stac3* mutant mice had fewer but larger myofibers

The total number of myofibers in the EDL muscle of newborn *Stac3* ^−/−^ mice was 26% or 18% less than that of wild-type or heterozygous littermates, respectively (*P*<0.05, Fig. 4B). However, the average cross-sectional area (CSA) of myofibers in the EDL muscle of *Stac3* ^−/−^ mice was 20% greater than that of wild-type or *Stac3* ^+/−^ mice (*P*<0.01, Fig. 4C). The myofibers in the EDL muscle of newborn wild-type or *Stac3* ^+/−^ mice had a normal distribution of CSA (Fig. 4D). Those in *Stac3* ^−/−^ mice, however, had a skewed-to-the right distribution (Fig. 4D). Despite these differences, the average size of the EDL muscle was not different between *Stac3* ^−/−^ mice and their wild-type or heterozygous littermates (*P*>0.1).

### Myofibers of *Stac3* mutant mice had fewer, smaller, and disorganized myofibrils

**Figure 5:**
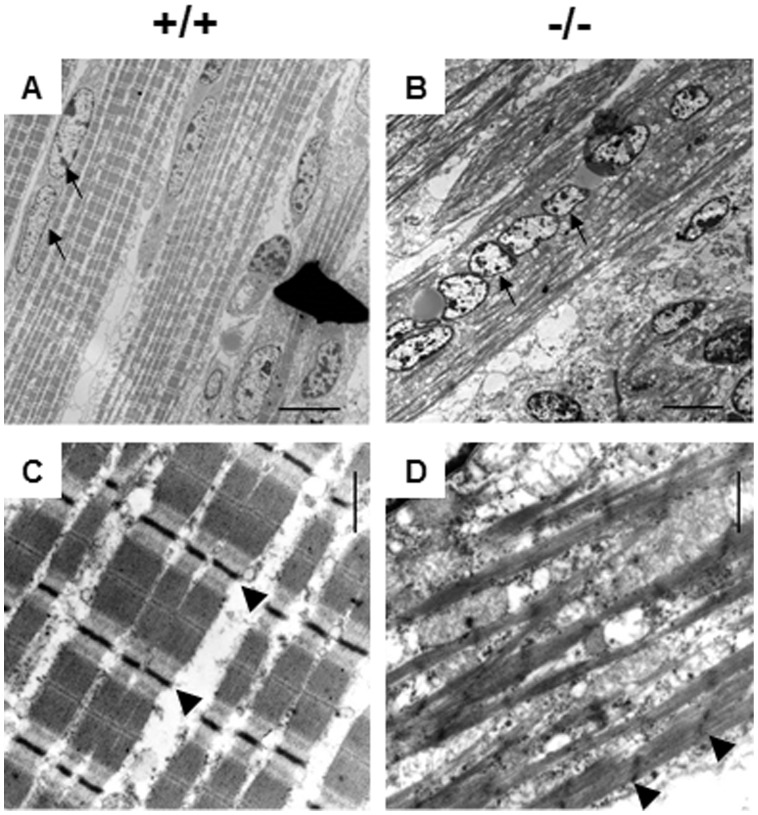
Representative electron micrographs of extensor digitorum longus (EDL) muscle of newborn *Stac3* ^−/−^ mice and *Stac3* ^+/+^ littermates. Note the differences in the location of myonuclei and the size and arrangement of myofibrils between *Stac3* homozygous mutant and wild-type muscles. Arrows point to myonuclei (micrographs A and B), and arrowheads indicate Z-lines (micrographs C and D). Scale bars  = 10 µm for micrographs A and B; Scale bars  = 500 nm for micrographs C and D.

We examined the ultrastructure of skeletal muscle with transmission electron microscopy (TEM). Myofibers in the EDL muscle of newborn wild-type mice had abundant, well-aligned, and near-uniform myofibrils and sarcomeres (Fig. 5A and 5C). In contrast, those of newborn *Stac3* ^−/−^ littermates had fewer, smaller, disarrayed, and sometimes fragmented myofibrils (Fig. 5B and 5D). Compared to sarcomeres in the EDL muscle of wild-type mice (Fig. 5C), those of *Stac3* ^−/−^ mice had streaming Z-lines (Fig. 5D). The TEM also confirmed the central location of myonuclei in newborn *Stac3* ^−/−^ mice (Fig. 5B) and the peripheral location of myonuclei in newborn wild-type mice (Fig. 5A).

## Discussion

In this study, we showed that the functionally unknown *Stac3* gene was specifically expressed in skeletal muscle, and we investigated the potential role of this gene in skeletal muscle development and function by disrupting its transcription in mice. All *Stac3* ^−*/*−^ mice were found dead at birth. This demonstrates that *Stac3* is essential to the development of viable mice. Whether at birth or dissected from the uterus, the *Stac3* ^−/−^ mice had an abnormal body curvature and dropping forelimbs, and did not respond to prodding. These phenotypes are similar to those caused by mutation of the myogenin gene [7], [8], the ryanodine receptor gene [17], and the dihydropyridine receptor gene [18], genes that are known to play a critical role in either the development of functional skeletal muscle or contraction of skeletal muscle. When a mouse is born, it becomes dependent on respiration for oxygen intake, which requires contraction of diaphragm and intercostal muscles [19], [20]. Therefore, it is likely that *Stac3* mutant mice died at birth because their dysfunctional diaphragm and intercostal muscles could not support breathing. Because *Stac3* is not expressed in the nervous system, it is unlikely that *Stac3* mutant mice die from dysfunctional neural control.

Histological analyses revealed that the skeletal muscle of newborn *Stac3* ^−/−^ mice had several abnormalities compared to that of wild-type mice, including: 1) most of their nuclei remained in the center of myofibers; 2) they had fewer yet larger myofibers; 3) they had a disproportional number of myofibers with large cross-sectional areas; 4) their myofibers had fewer, smaller, and less organized myofibrils. With these abnormalities, a skeletal muscle is unlikely able to function. Among these abnormalities, the most striking was that most of the myonuclei were located in the center of myofibers at a developmental stage (i.e. birth) when they should have been relocated to the periphery of myofibers [21]. While having centrally located nuclei is typically indicative of regeneration in adult skeletal muscle [5], [22], it is unlikely that skeletal muscle in fetal *Stac3* ^−*/*−^ mice was undergoing regeneration because every skeletal muscle examined had centrally located nuclei. Translocation of myonuclei from the center to the periphery is the hallmark of myofiber maturation during late embryonic development [21]. Therefore, it is possible that *Stac3* gene mutation disrupted myofiber maturation. This possibility is also supported by the observation that *Stac3* ^−/−^ myofibers had fewer and smaller myofibrils than wild-type myofibers at the same developmental stage. It is also possible that *Stac3* disruption disrupted the ill-defined mechanism that controls the translocation of myonuclei during skeletal muscle development.

Myofibers of *Stac3* ^−*/*−^ mice had greater cross-sectional areas than those of wild-type or *Stac3* ^+/−^ littermates. This difference was probably not due to myofiber hypertrophy in *Stac3* ^−/−^ mice because myofibers in those mice indeed had fewer and smaller myofibrils than in wild-type or *Stac3* ^+/−^. The difference was more likely caused by increased fusion of myoblasts into myotubes in *Stac3* mutant mouse embryos. The relative numbers of proliferating myoblasts versus differentiating myoblasts during muscle development can determine the total number of myofibers formed in a mature muscle [23], [24]. If myoblasts withdraw from the cell cycle and begin differentiation before an adequate number of founder myoblasts have developed, this would decrease the overall number of myofibers formed. Therefore, an increase in myoblast fusion could also explain why a *Stac3* ^−*/*−^ skeletal muscle had fewer myofibers than a wild-type or a *Stac3* ^+/−^ skeletal muscle. Myogenesis is a highly orchestrated program, and premature myoblast fusion could lead to the formation of dysfunctional muscle [25], [26]. Therefore, increased fusion of myoblasts may also explain why skeletal muscle in *Stac3* mutant mice was unable to contract.

Unlike the master regulators of skeletal muscle development, the MRFs (MyoD, myogenin, MRF4, Myf5), Stac3 is not a transcription factor and does not appear to have direct DNA-binding ability. The Stac proteins are believed to be confined to the cytosolic domain of the cell with no evidence for enzymatic activity [15], [27]. However, Stac3 does possess two domains (SH3 and C1) that are frequent components of signaling proteins [16], [28]. Interestingly, two known regulators of myoblast fusion, Mbc and DCrk, also contain the SH3 domain [29], [30]. Therefore, Stac3 might be involved in the process of myoblast fusion through the SH3 domain. However, both the SH3 domain and the C1 domain can interact with multiple partners in a cell [29], [30]. The multiple skeletal muscle phenotypes caused by *Stac3* gene disruption may be mediated by different Stac3-protein interactions.

In summary, we have demonstrated that the *Stac3* gene is exclusively expressed in skeletal muscle and that it is essential for development of functional skeletal muscle and mice viable at birth. The domain structures of Stac3 protein suggest that it is a signaling protein, and further investigation into the molecular and cellular cause of abnormalities in *Stac3* mutant skeletal muscle may reveal novel signaling mechanisms that regulate skeletal muscle development or function. The centrally located myonuclei and lack of muscle contraction phenotypes of *Stac3* mutant mice are hallmarks of centronuclear/myotubular myopathies in humans [31], [32]. Therefore, the *Stac3* mutant mouse may represent a useful animal model for understanding the pathophysiology of this muscle disease. While this manuscript was being prepared, a study published in *JBC* indicates that *Stac3* is essential for myotube formation in zebrafish [33]. Our study, however, shows that *Stac3* gene inactivation does not prevent myotube formation in mice (Fig. 3, Fig. 5). The reason for this difference is not clear, but it may be related to the use of different model systems between the two studies.

## References

[1] Rudnicki MA, Braun T, Hinuma S, Jaenisch R (1992) Inactivation of MyoD in Mice Leads to up-Regulation of the Myogenic HLH Gene Myf-5 and Results in Apparently Normal Muscle Development. Cell 71: 383–390. 1330322 10.1016/0092-8674(92)90508-a

[2] Braun T, Buschhausen-Denker G, Bober E, Tannich E, Arnold HH (1989) A novel human muscle factor related to but distinct from MyoD1 induces myogenic conversion in 10T1/2 fibroblasts. EMBO J 8: 701–709. 2721498 10.1002/j.1460-2075.1989.tb03429.x PMC400865

[3] Wright WE, Sassoon DA, Lin VK (1989) Myogenin, a factor regulating myogenesis, has a domain homologous to MyoD. Cell 56: 607–617. 2537150 10.1016/0092-8674(89)90583-7

[4] Rhodes SJ, Konieczny SF (1989) Identification of MRF4: a new member of the muscle regulatory factor gene family. Genes Dev 3: 2050–2061. 2560751 10.1101/gad.3.12b.2050

[5] Charge SB, Rudnicki MA (2004) Cellular and molecular regulation of muscle regeneration. Physiological Reviews 84: 209–238. 14715915 10.1152/physrev.00019.2003

[6] Rudnicki MA, Schnegelsberg PNJ, Stead RH, Braun T, Arnold HH, et al (1993) MyoD or Myf-5 Is Required for the Formation of Skeletal-Muscle. Cell 75: 1351–1359. 8269513 10.1016/0092-8674(93)90621-v

[7] Nabeshima Y, Hanaoka K, Hayasaka M, Esumi E, Li S, et al (1993) Myogenin gene disruption results in perinatal lethality because of severe muscle defect. Nature 364: 532–535. 8393146 10.1038/364532a0

[8] Hasty P, Bradley A, Morris JH, Edmondson DG, Venuti JM, et al (1993) Muscle deficiency and neonatal death in mice with a targeted mutation in the myogenin gene. Nature 364: 501–506. 8393145 10.1038/364501a0

[9] Yoon JK, Olson EN, Arnold HH, Wold BJ (1997) Different MRF4 knockout alleles differentially disrupt Myf-5 expression: cis-regulatory interactions at the MRF4/Myf-5 locus. Dev Biol 188: 349–362. 9268580 10.1006/dbio.1997.8670

[10] Yanai I, Benjamin H, Shmoish M, Chalifa-Caspi V, Shklar M, et al (2005) Genome-wide midrange transcription profiles reveal expression level relationships in human tissue specification. Bioinformatics 21: 650–659. 15388519 10.1093/bioinformatics/bti042

[11] Kawai J, Suzuki H, Hara A, Hirose K, Watanabe S (1998) Human and mouse chromosomal mapping of Stac, a neuron-specific protein with an SH3 domain. Genomics 47: 140–142. 9465308 10.1006/geno.1997.5107

[12] Legha W, Gaillard S, Gascon E, Malapert P, Hocine M, et al (2010) stac1 and stac2 genes define discrete and distinct subsets of dorsal root ganglia neurons. Gene Expr Patterns 10: 368–375. 20736085 10.1016/j.gep.2010.08.003

[13] Skarnes WC, Rosen B, West AP, Koutsourakis M, Bushell W, et al (2011) A conditional knockout resource for the genome-wide study of mouse gene function. Nature 474: 337–342. 21677750 10.1038/nature10163 PMC3572410

[14] Buchberger A, Nomokonova N, Arnold HH (2003) Myf5 expression in somites and limb buds of mouse embryos is controlled by two distinct distal enhancer activities. Development 130: 3297–3307. 12783799 10.1242/dev.00557

[15] Abrams CS, Zhao W (1995) SH3 domains specifically regulate kinase activity of expressed Src family proteins. Journal of Biological Chemistry 270: 333–339. 7529230 10.1074/jbc.270.1.333

[16] Colon-Gonzalez F, Kazanietz MG (2006) C1 domains exposed: from diacylglycerol binding to protein-protein interactions. Biochim Biophys Acta 1761: 827–837. 16861033 10.1016/j.bbalip.2006.05.001

[17] Takeshima H, Iino M, Takekura H, Nishi M, Kuno J, et al (1994) Excitation-contraction uncoupling and muscular degeneration in mice lacking functional skeletal muscle ryanodine-receptor gene. Nature 369: 556–559. 7515481 10.1038/369556a0

[18] Pai AC (1965) Developmental Genetics of a Lethal Mutation, Muscular Dysgenesis (Mdg), in the Mouse. I. Genetic Analysis and Gross Morphology. Developmental Biology 11: 82–92. 14300095 10.1016/0012-1606(65)90038-2

[19] Copp AJ (1995) Death before birth: clues from gene knockouts and mutations. Trends in Genetics 11: 87–93. 7732578 10.1016/S0168-9525(00)89008-3

[20] Turgeon B, Meloche S (2009) Interpreting neonatal lethal phenotypes in mouse mutants: insights into gene function and human diseases. Physiological Reviews 89: 1–26. 19126753 10.1152/physrev.00040.2007

[21] Ontell M, Kozeka K (1984) The organogenesis of murine striated muscle: a cytoarchitectural study. American journal of anatomy 171: 133–148. 6496372 10.1002/aja.1001710202

[22] Mitchell CA, McGeachie JK, Grounds MD (1992) Cellular differences in the regeneration of murine skeletal muscle: a quantitative histological study in SJL/J and BALB/c mice. Cell and Tissue Research 269: 159–166. 1423478 10.1007/BF00384736

[23] Dlugosz AA, Tapscott SJ, Holtzer H (1983) Effects of phorbol 12-myristate 13-acetate on the differentiation program of embryonic chick skeletal myoblasts. Cancer Research 43: 2780–2789. 6342759

[24] Atreya KB, Fernandes JJ (2008) Founder cells regulate fiber number but not fiber formation during adult myogenesis in Drosophila. Developmental Biology 321: 123–140. 18616937 10.1016/j.ydbio.2008.06.023

[25] Bentzinger CF, Wang YX, Rudnicki MA (2012) Building muscle: molecular regulation of myogenesis. Cold Spring Harb Perspect Biol 4. 10.1101/cshperspect.a008342 PMC3281568 22300977

[26] Schuster-Gossler K, Cordes R, Gossler A (2007) Premature myogenic differentiation and depletion of progenitor cells cause severe muscle hypotrophy in Delta1 mutants. Proceedings of the National Academy of Science of the USA 104: 537–542. 10.1073/pnas.0608281104 PMC1766420 17194759

[27] Suzuki H, Kawai J, Taga C, Yaoi T, Hara A, et al (1996) Stac, a novel neuron-specific protein with cysteine-rich and SH3 domains. Biochem Biophys Res Commun 229: 902–909. 8954993 10.1006/bbrc.1996.1900

[28] Mayer BJ (2001) SH3 domains: complexity in moderation. Journal of Cell Science 114: 1253–1263. 11256992 10.1242/jcs.114.7.1253

[29] Dworak HA, Sink H (2002) Myoblast fusion in Drosophila. Bioessays 24: 591–601. 12111720 10.1002/bies.10115

[30] Chen EH, Olson EN (2004) Towards a molecular pathway for myoblast fusion in Drosophila. Trends Cell Biol 14: 452–460. 15308212 10.1016/j.tcb.2004.07.008

[31] Jungbluth H, Wallgren-Pettersson C, Laporte J (2008) Centronuclear (myotubular) myopathy. Orphanet J Rare Dis 3: 26. 18817572 10.1186/1750-1172-3-26 PMC2572588

[32] Romero NB (2010) Centronuclear myopathies: a widening concept. Neuromuscular Disorders 20: 223–228. 20181480 10.1016/j.nmd.2010.01.014

[33] Bower NI, de la Serrana DG, Cole NJ, Hollway GE, Lee HT, et al (2012) Stac3 is required for myotube formation and myogenic differentiation in vertebrate skeletal muscle. Journal of Biological Chemistry 287: 43936–43949. 23076145 10.1074/jbc.M112.361311 PMC3527977

